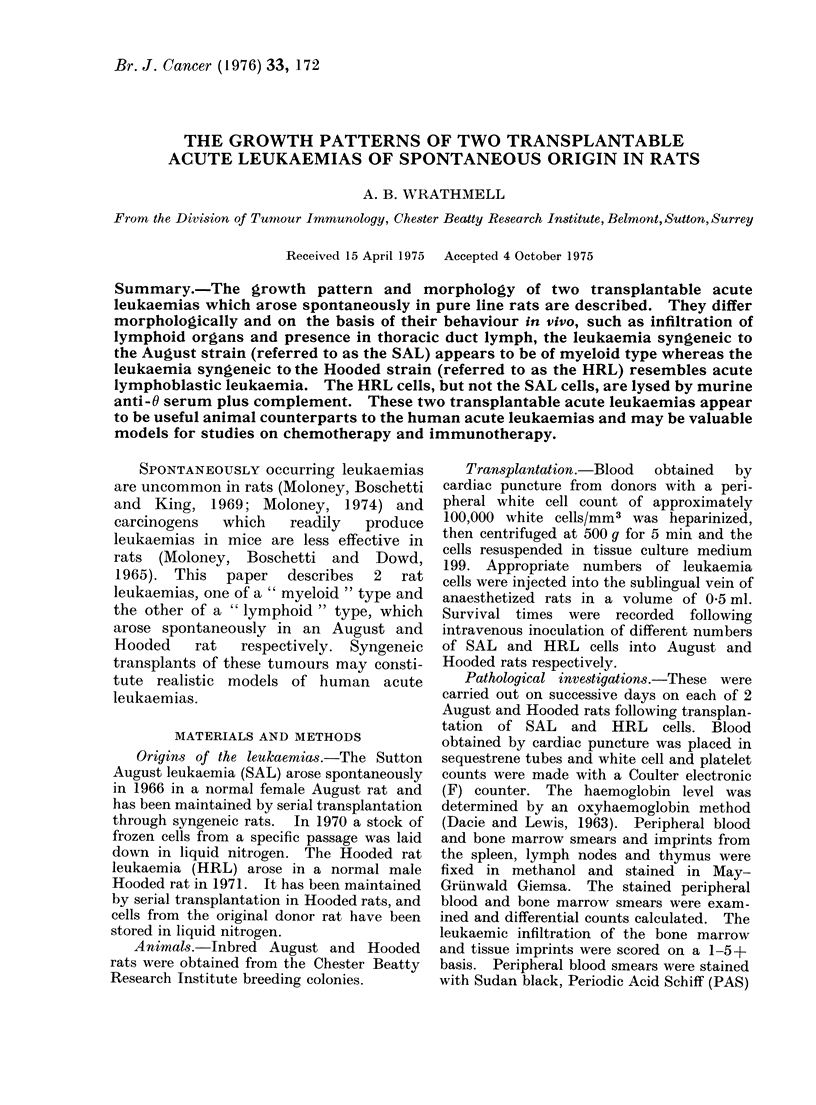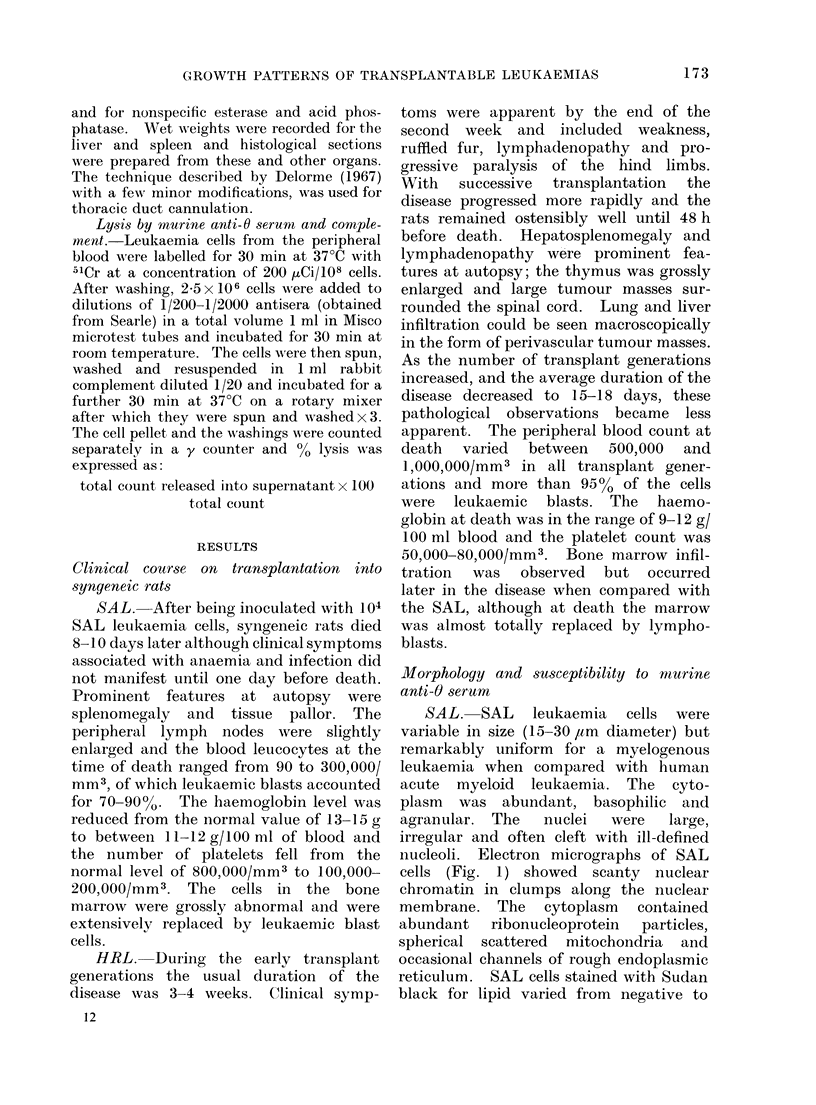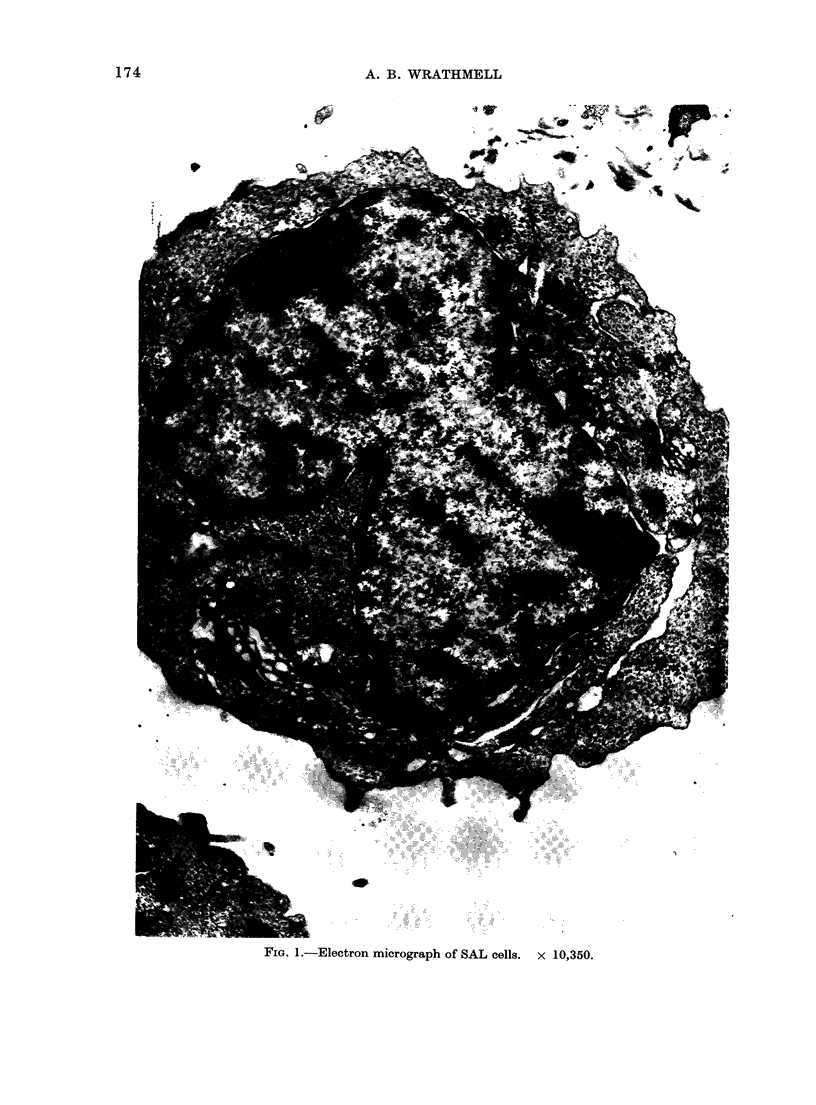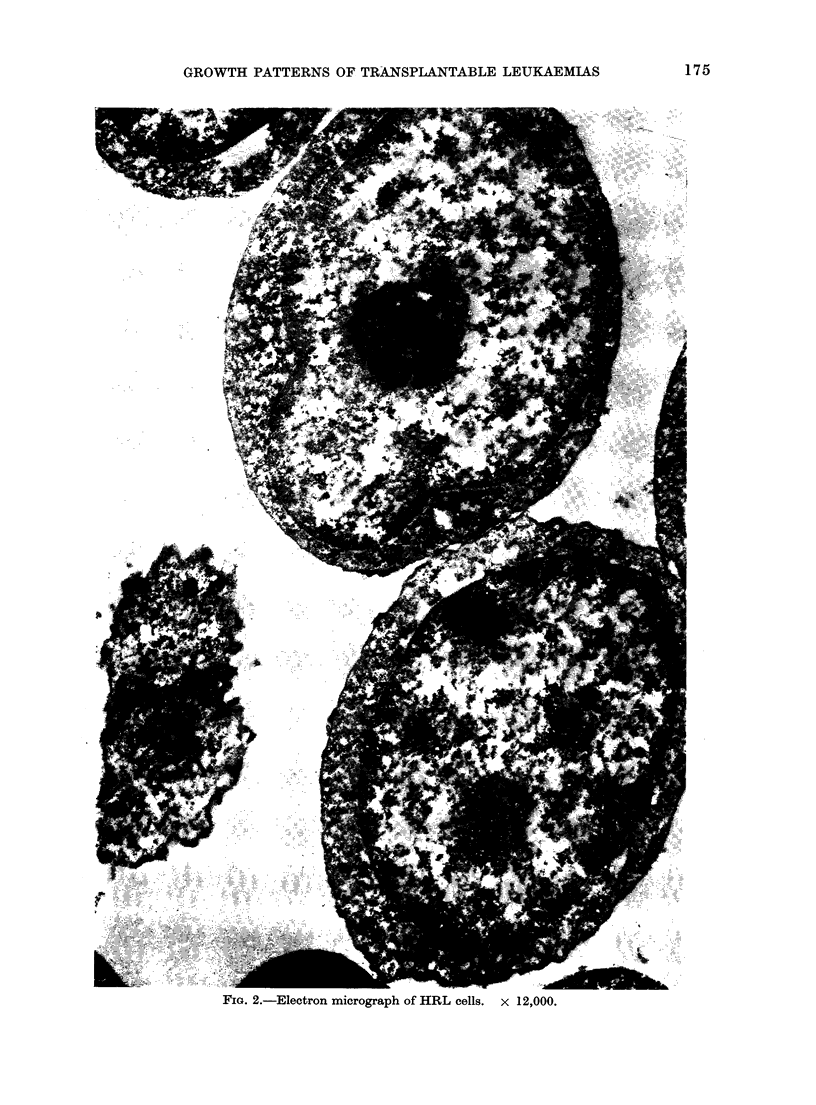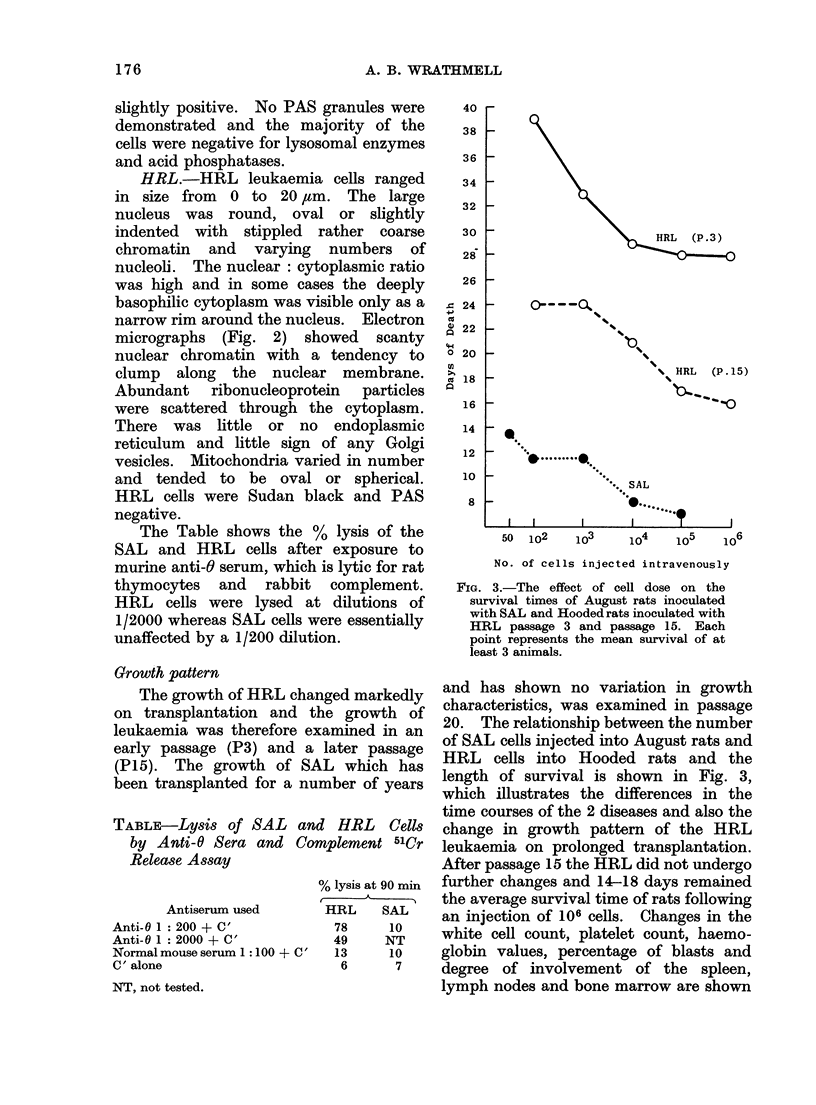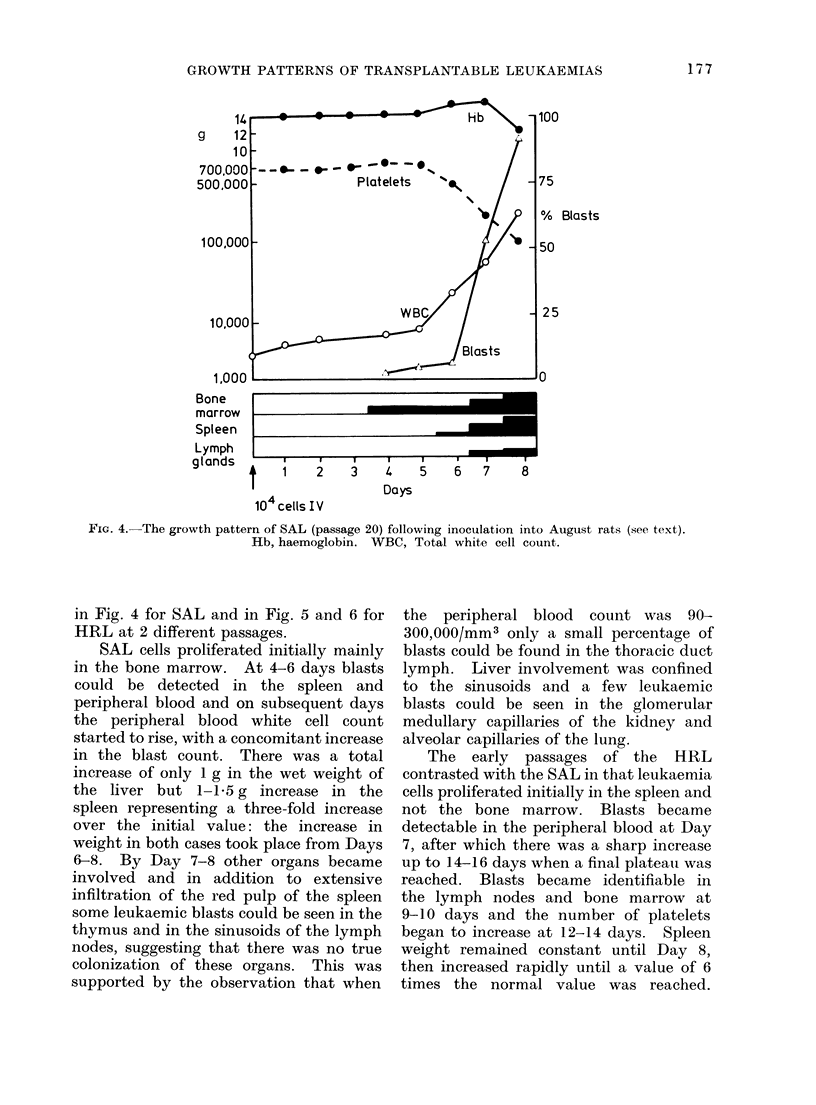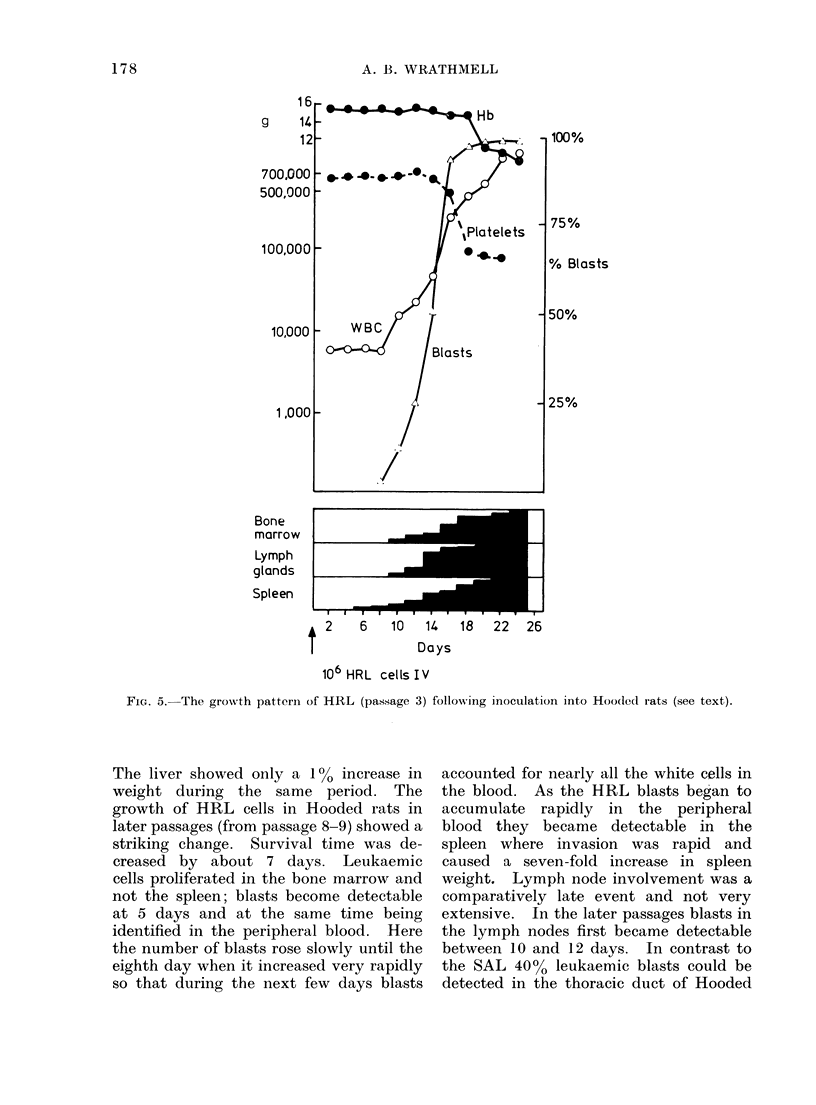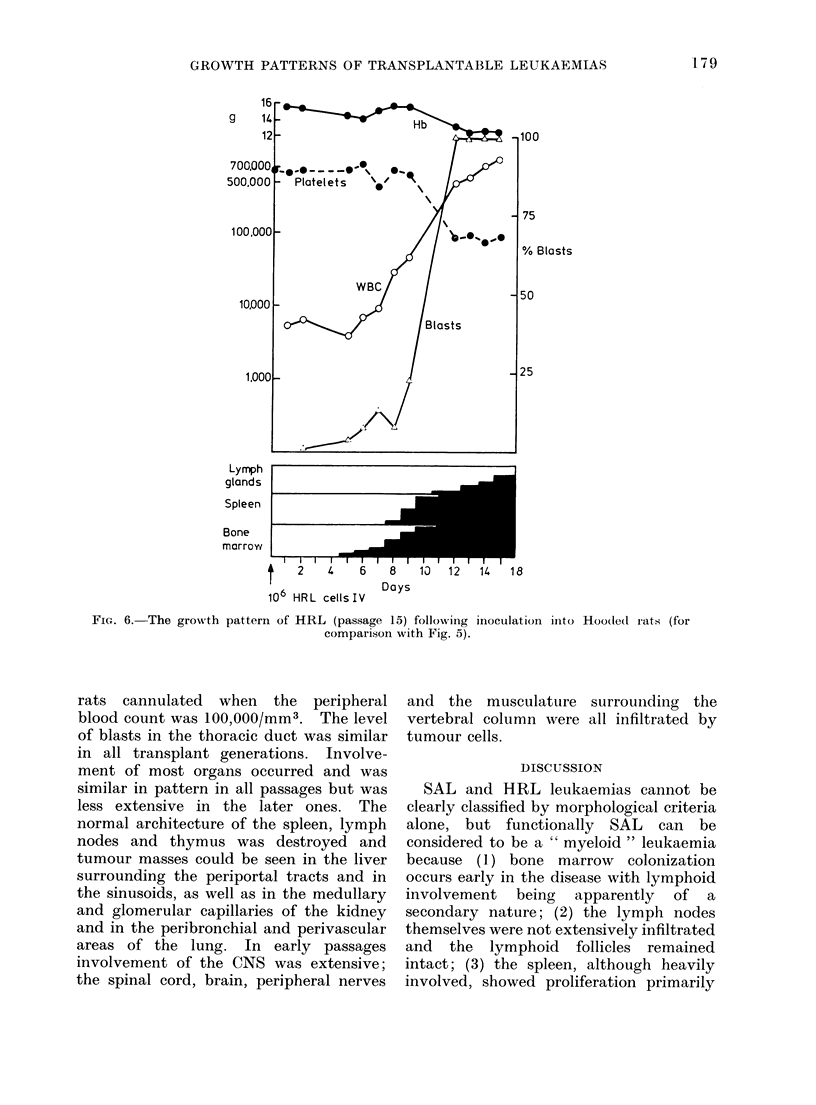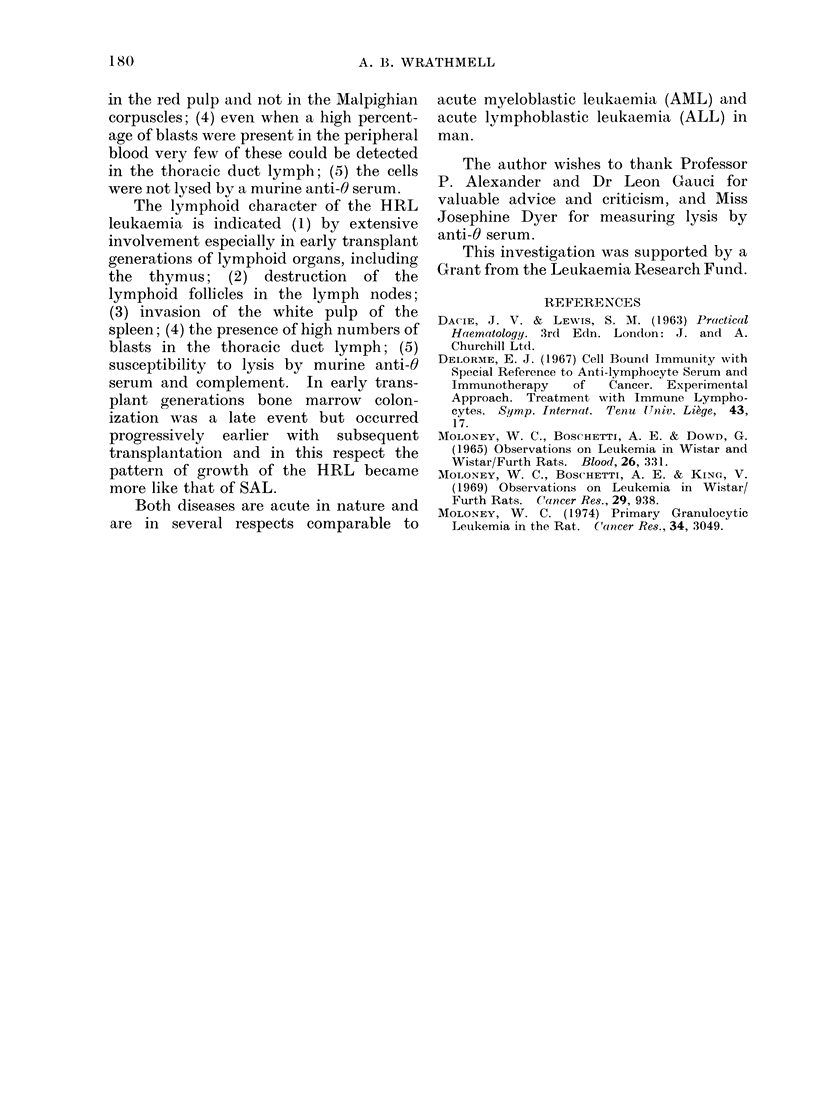# The growth patterns of two transplantable acute leukaemias of spontaneous origin in rats.

**DOI:** 10.1038/bjc.1976.22

**Published:** 1976-02

**Authors:** A. B. Wrathmell

## Abstract

**Images:**


*Br. J. Cancer* (1976) **33**, 172

### THE GROWTH PATTERNS OF TWO TRANSPLANTABLE ACUTE LEUKAEMIAS OF SPONTANEOUS ORIGIN IN RATS

A. B. WRATHMELL

*From the Division of Tumour Immunology, Chester Beatty Research Institute, Belmont, Sutton, Surrey*

Received 15 April 1975 Accepted 4 October 1975

**Summary.—The growth pattern and morphology of two transplantable acute leukaemias which arose spontaneously in pure line rats are described. They differ morphologically and on the basis of their behaviour *in vivo*, such as infiltration of lymphoid organs and presence in thoracic duct lymph, the leukaemia syngeneic to the August strain (referred to as the SAL) appears to be of myeloid type whereas the leukaemia syngeneic to the Hooded strain (referred to as the HRL) resembles acute lymphoblastic leukaemia. The HRL cells, but not the SAL cells, are lysed by murine anti-$\theta$ serum plus complement. These two transplantable acute leukaemias appear to be useful animal counterparts to the human acute leukaemias and may be valuable models for studies on chemotherapy and immunotherapy.**

SPONTANEOUSLY occurring leukaemias are uncommon in rats (Moloney, Boschetti and King, 1969; Moloney, 1974) and carcinogens which readily produce leukaemias in mice are less effective in rats (Moloney, Boschetti and Dowd, 1965). This paper describes 2 rat leukaemias, one of a " myeloid " type and the other of a " lymphoid " type, which arose spontaneously in an August and Hooded rat respectively. Syngeneic transplants of these tumours may constitute realistic models of human acute leukaemias.

### MATERIALS AND METHODS

*Origins of the leukaemias.*—The Sutton August leukaemia (SAL) arose spontaneously in 1966 in a normal female August rat and has been maintained by serial transplantation through syngeneic rats. In 1970 a stock of frozen cells from a specific passage was laid down in liquid nitrogen. The Hooded rat leukaemia (HRL) arose in a normal male Hooded rat in 1971. It has been maintained by serial transplantation in Hooded rats, and cells from the original donor rat have been stored in liquid nitrogen.

*Animals.*—Inbred August and Hooded rats were obtained from the Chester Beatty Research Institute breeding colonies.

*Transplantation.*—Blood obtained by cardiac puncture from donors with a peripheral white cell count of approximately 100,000 white cells/mm$^3$ was heparinized, then centrifuged at 500 $g$ for 5 min and the cells resuspended in tissue culture medium 199. Appropriate numbers of leukaemia cells were injected into the sublingual vein of anaesthetized rats in a volume of 0·5 ml. Survival times were recorded following intravenous inoculation of different numbers of SAL and HRL cells into August and Hooded rats respectively.

*Pathological investigations.*—These were carried out on successive days on each of 2 August and Hooded rats following transplantation of SAL and HRL cells. Blood obtained by cardiac puncture was placed in sequestrene tubes and white cell and platelet counts were made with a Coulter electronic (F) counter. The haemoglobin level was determined by an oxyhaemoglobin method (Dacie and Lewis, 1963). Peripheral blood and bone marrow smears and imprints from the spleen, lymph nodes and thymus were fixed in methanol and stained in May-Grünwald Giemsa. The stained peripheral blood and bone marrow smears were examined and differential counts calculated. The leukaemic infiltration of the bone marrow and tissue imprints were scored on a 1–5+ basis. Peripheral blood smears were stained with Sudan black, Periodic Acid Schiff (PAS)

and for nonspecific esterase and acid phosphatase. Wet weights were recorded for the liver and spleen and histological sections were prepared from these and other organs. The technique described by Delorme (1967) with a few minor modifications, was used for thoracic duct cannulation.

*Lysis by murine anti-θ serum and complement.*—Leukaemia cells from the peripheral blood were labelled for 30 min at 37°C with $^{51}Cr$ at a concentration of 200 $\mu$Ci/$10^8$ cells. After washing, $2 \cdot 5 \times 10^6$ cells were added to dilutions of 1/200–1/2000 antisera (obtained from Searle) in a total volume 1 ml in Misco microtest tubes and incubated for 30 min at room temperature. The cells were then spun, washed and resuspended in 1 ml rabbit complement diluted 1/20 and incubated for a further 30 min at 37°C on a rotary mixer after which they were spun and washed × 3. The cell pellet and the washings were counted separately in a $\gamma$ counter and % lysis was expressed as:

$$\frac{\text{total count released into supernatant} \times 100}{\text{total count}}$$

### RESULTS

### *Clinical course on transplantation into syngeneic rats*

*SAL.*—After being inoculated with $10^4$ SAL leukaemia cells, syngeneic rats died 8–10 days later although clinical symptoms associated with anaemia and infection did not manifest until one day before death. Prominent features at autopsy were splenomegaly and tissue pallor. The peripheral lymph nodes were slightly enlarged and the blood leucocytes at the time of death ranged from 90 to 300,000/mm$^3$, of which leukaemic blasts accounted for 70–90%. The haemoglobin level was reduced from the normal value of 13–15 g to between 11–12 g/100 ml of blood and the number of platelets fell from the normal level of 800,000/mm$^3$ to 100,000–200,000/mm$^3$. The cells in the bone marrow were grossly abnormal and were extensively replaced by leukaemic blast cells.

*HRL.*—During the early transplant generations the usual duration of the disease was 3–4 weeks. Clinical symptoms were apparent by the end of the second week and included weakness, ruffled fur, lymphadenopathy and progressive paralysis of the hind limbs. With successive transplantation the disease progressed more rapidly and the rats remained ostensibly well until 48 h before death. Hepatosplenomegaly and lymphadenopathy were prominent features at autopsy; the thymus was grossly enlarged and large tumour masses surrounded the spinal cord. Lung and liver infiltration could be seen macroscopically in the form of perivascular tumour masses. As the number of transplant generations increased, and the average duration of the disease decreased to 15–18 days, these pathological observations became less apparent. The peripheral blood count at death varied between 500,000 and 1,000,000/mm$^3$ in all transplant generations and more than 95% of the cells were leukaemic blasts. The haemoglobin at death was in the range of 9–12 g/100 ml blood and the platelet count was 50,000–80,000/mm$^3$. Bone marrow infiltration was observed but occurred later in the disease when compared with the SAL, although at death the marrow was almost totally replaced by lymphoblasts.

### *Morphology and susceptibility to murine anti-θ serum*

*SAL.*—SAL leukaemia cells were variable in size (15–30 $\mu$m diameter) but remarkably uniform for a myelogenous leukaemia when compared with human acute myeloid leukaemia. The cytoplasm was abundant, basophilic and agranular. The nuclei were large, irregular and often cleft with ill-defined nucleoli. Electron micrographs of SAL cells (Fig. 1) showed scanty nuclear chromatin in clumps along the nuclear membrane. The cytoplasm contained abundant ribonucleoprotein particles, spherical scattered mitochondria and occasional channels of rough endoplasmic reticulum. SAL cells stained with Sudan black for lipid varied from negative to

FIG. 1.—Electron micrograph of SAL cells. × 10,350.

FIG. 2.—Electron micrograph of HRL cells. × 12,000.

slightly positive. No PAS granules were demonstrated and the majority of the cells were negative for lysosomal enzymes and acid phosphatases.

*HRL*.—HRL leukaemia cells ranged in size from 0 to 20 $\mu$m. The large nucleus was round, oval or slightly indented with stippled rather coarse chromatin and varying numbers of nucleoli. The nuclear : cytoplasmic ratio was high and in some cases the deeply basophilic cytoplasm was visible only as a narrow rim around the nucleus. Electron micrographs (Fig. 2) showed scanty nuclear chromatin with a tendency to clump along the nuclear membrane. Abundant ribonucleoprotein particles were scattered through the cytoplasm. There was little or no endoplasmic reticulum and little sign of any Golgi vesicles. Mitochondria varied in number and tended to be oval or spherical. HRL cells were Sudan black and PAS negative.

The Table shows the % lysis of the SAL and HRL cells after exposure to murine anti-$\theta$ serum, which is lytic for rat thymocytes and rabbit complement. HRL cells were lysed at dilutions of 1/2000 whereas SAL cells were essentially unaffected by a 1/200 dilution.

*Growth pattern*

The growth of HRL changed markedly on transplantation and the growth of leukaemia was therefore examined in an early passage (P3) and a later passage (P15). The growth of SAL which has been transplanted for a number of years and has shown no variation in growth characteristics, was examined in passage 20. The relationship between the number of SAL cells injected into August rats and HRL cells into Hooded rats and the length of survival is shown in Fig. 3, which illustrates the differences in the time courses of the 2 diseases and also the change in growth pattern of the HRL leukaemia on prolonged transplantation. After passage 15 the HRL did not undergo further changes and 14–18 days remained the average survival time of rats following an injection of $10^6$ cells. Changes in the white cell count, platelet count, haemoglobin values, percentage of blasts and degree of involvement of the spleen, lymph nodes and bone marrow are shown

FIG. 3.—The effect of cell dose on the survival times of August rats inoculated with SAL and Hooded rats inoculated with HRL passage 3 and passage 15. Each point represents the mean survival of at least 3 animals.

TABLE—*Lysis of SAL and HRL Cells by Anti-θ Sera and Complement* $^{51}$*Cr Release Assay*

| Antiserum used | % lysis at 90 min HRL | SAL |
|---|---|---|
| Anti-$\theta$ 1 : 200 + C′ | 78 | 10 |
| Anti-$\theta$ 1 : 2000 + C′ | 49 | NT |
| Normal mouse serum 1 : 100 + C′ | 13 | 10 |
| C′ alone | 6 | 7 |

NT, not tested.

FIG. 4.—The growth pattern of SAL (passage 20) following inoculation into August rats (see text). Hb, haemoglobin. WBC, Total white cell count.

in Fig. 4 for SAL and in Fig. 5 and 6 for HRL at 2 different passages.

SAL cells proliferated initially mainly in the bone marrow. At 4–6 days blasts could be detected in the spleen and peripheral blood and on subsequent days the peripheral blood white cell count started to rise, with a concomitant increase in the blast count. There was a total increase of only 1 g in the wet weight of the liver but 1–1·5 g increase in the spleen representing a three-fold increase over the initial value: the increase in weight in both cases took place from Days 6–8. By Day 7–8 other organs became involved and in addition to extensive infiltration of the red pulp of the spleen some leukaemic blasts could be seen in the thymus and in the sinusoids of the lymph nodes, suggesting that there was no true colonization of these organs. This was supported by the observation that when the peripheral blood count was 90–300,000/$mm^3$ only a small percentage of blasts could be found in the thoracic duct lymph. Liver involvement was confined to the sinusoids and a few leukaemic blasts could be seen in the glomerular medullary capillaries of the kidney and alveolar capillaries of the lung.

The early passages of the HRL contrasted with the SAL in that leukaemia cells proliferated initially in the spleen and not the bone marrow. Blasts became detectable in the peripheral blood at Day 7, after which there was a sharp increase up to 14–16 days when a final plateau was reached. Blasts became identifiable in the lymph nodes and bone marrow at 9–10 days and the number of platelets began to increase at 12–14 days. Spleen weight remained constant until Day 8, then increased rapidly until a value of 6 times the normal value was reached.

FIG. 5.—The growth pattern of HRL (passage 3) following inoculation into Hooded rats (see text).

The liver showed only a 1% increase in weight during the same period. The growth of HRL cells in Hooded rats in later passages (from passage 8–9) showed a striking change. Survival time was decreased by about 7 days. Leukaemic cells proliferated in the bone marrow and not the spleen; blasts become detectable at 5 days and at the same time being identified in the peripheral blood. Here the number of blasts rose slowly until the eighth day when it increased very rapidly so that during the next few days blasts accounted for nearly all the white cells in the blood. As the HRL blasts began to accumulate rapidly in the peripheral blood they became detectable in the spleen where invasion was rapid and caused a seven-fold increase in spleen weight. Lymph node involvement was a comparatively late event and not very extensive. In the later passages blasts in the lymph nodes first became detectable between 10 and 12 days. In contrast to the SAL 40% leukaemic blasts could be detected in the thoracic duct of Hooded

FIG. 6.—The growth pattern of HRL (passage 15) following inoculation into Hooded rats (for comparison with Fig. 5).

rats cannulated when the peripheral blood count was 100,000/mm$^3$. The level of blasts in the thoracic duct was similar in all transplant generations. Involvement of most organs occurred and was similar in pattern in all passages but was less extensive in the later ones. The normal architecture of the spleen, lymph nodes and thymus was destroyed and tumour masses could be seen in the liver surrounding the periportal tracts and in the sinusoids, as well as in the medullary and glomerular capillaries of the kidney and in the peribronchial and perivascular areas of the lung. In early passages involvement of the CNS was extensive; the spinal cord, brain, peripheral nerves and the musculature surrounding the vertebral column were all infiltrated by tumour cells.

### DISCUSSION

SAL and HRL leukaemias cannot be clearly classified by morphological criteria alone, but functionally SAL can be considered to be a " myeloid " leukaemia because (1) bone marrow colonization occurs early in the disease with lymphoid involvement being apparently of a secondary nature; (2) the lymph nodes themselves were not extensively infiltrated and the lymphoid follicles remained intact; (3) the spleen, although heavily involved, showed proliferation primarily in the red pulp and not in the Malpighian corpuscles; (4) even when a high percentage of blasts were present in the peripheral blood very few of these could be detected in the thoracic duct lymph; (5) the cells were not lysed by a murine anti-$\theta$ serum.

The lymphoid character of the HRL leukaemia is indicated (1) by extensive involvement especially in early transplant generations of lymphoid organs, including the thymus; (2) destruction of the lymphoid follicles in the lymph nodes; (3) invasion of the white pulp of the spleen; (4) the presence of high numbers of blasts in the thoracic duct lymph; (5) susceptibility to lysis by murine anti-$\theta$ serum and complement. In early transplant generations bone marrow colonization was a late event but occurred progressively earlier with subsequent transplantation and in this respect the pattern of growth of the HRL became more like that of SAL.

Both diseases are acute in nature and are in several respects comparable to acute myeloblastic leukaemia (AML) and acute lymphoblastic leukaemia (ALL) in man.

The author wishes to thank Professor P. Alexander and Dr Leon Gauci for valuable advice and criticism, and Miss Josephine Dyer for measuring lysis by anti-$\theta$ serum.

This investigation was supported by a Grant from the Leukaemia Research Fund.

### REFERENCES

DACIE, J. V. & LEWIS, S. M. (1963) *Practical Haematology*. 3rd Edn. London: J. and A. Churchill Ltd.

DELORME, E. J. (1967) Cell Bound Immunity with Special Reference to Anti-lymphocyte Serum and Immunotherapy of Cancer. Experimental Approach. Treatment with Immune Lymphocytes. *Symp. Internat. Tenu Univ. Liège*, **43**, 17.

MOLONEY, W. C., BOSCHETTI, A. E. & DOWD, G. (1965) Observations on Leukemia in Wistar and Wistar/Furth Rats. *Blood*, **26**, 331.

MOLONEY, W. C., BOSCHETTI, A. E. & KING, V. (1969) Observations on Leukemia in Wistar/Furth Rats. *Cancer Res.*, **29**, 938.

MOLONEY, W. C. (1974) Primary Granulocytic Leukemia in the Rat. *Cancer Res.*, **34**, 3049.